# Seroprevalence of SARS-CoV-2 antibodies in healthcare workers at a tertiary care hospital in Riyadh, Saudi Arabia

**DOI:** 10.1016/j.ijregi.2021.11.009

**Published:** 2021-11-29

**Authors:** Reem S. Almaghrabi, Osamah I. Alsagheir, Rawan M. Alquaiz, Othman Z. Alhekail, Abdulrahman M. Abaalkhail, Atheer A. Alduaij, Ghadah F. Algwaiz, Morad A. Alkaff, Sahar I. Althawadi

**Affiliations:** 1 Department of Medicine, King Faisal Hospital and Research Centre, Riyadh, Saudi Arabia; 2 Microbiology Laboratory, Department of Pathology and Laboratory Medicine, King Faisal Hospital and Research Centre, Riyadh, Saudi Arabia

**Keywords:** SARS-CoV-2, COVID-19, antibodies, prevalence, infection, asymptomatic

## Abstract

**Background:**

The SARS-CoV-2 pandemic has led to a strain on medical resources. The development of countermeasures to prevent its spread is evolving. Healthcare workers (HCWs) are at high risk for contracting and transmitting the disease.

**Methods:**

Serology testing of volunteer HCWs was performed at King Faisal Specialist Hospital and Research Center in Riyadh (the Center) in order to determine the prevalence of SARS-CoV-2 antibodies, as well as the associated risk factors, in the hope of implementing adequate prevention and control measures.

**Results:**

1076 subjects participated in this study, of whom 24.3% were seropositive. The majority were nurses (379, 35%) or physicians (245, 22.2%). 392 (36.4%) of the 1076 subjects were caregivers for COVID-19 patients, and 463 (43.0%) reported contact with infected employees. There was a statistically significant association between taking care of COVID-19 patients and being diagnosed with COVID-19 (chi-square test, *p* = 0.046). There was a significant association between being in contact with infected employees and having a positive IgG result (chi-square test, *p* < 0.001).

**Conclusions:**

A baseline analysis of SARS-CoV-2 seropositivity in HCWs at a large tertiary care hospital in Riyadh was performed as the first part of a prospective study of HCWs. The reported seropositivity was 24.3% — higher than that of other hospitals in Riyadh. IgG testing was very useful in the detection of previous SARS-CoV-2 infection, as it has high negative predictive value.

## Highlights

• Total seroprevalence of SARS-CoV-2 antibodies was 24.3%; 3.6% were asymptomatic
• IgG testing had a high negative predictive value in diagnosing COVID-19 disease
• Contact with COVID-19 patients found to be linked to asymptomatic COVID-19 diagnosis in HCWs
• Strong association between contact with infected employee and having a positive IgG test

## Introduction

The novel coronavirus (SARS-CoV-2) that causes COVID-19 disease was first detected in Wuhan, China in December 2019. After its discovery, the number of cases rose rapidly, leading to an international health concern. On March 11, 2020, the World Health Organization officially declared COVID-19 a global pandemic. At the time of writing, the rapid spread of the infection had resulted in a total of 127 million cases and 2.8 million deaths worldwide (Dong et al., 2020). Around 388 000 cases had been detected in Saudi Arabia as of March 2021 (Dong et al., 2020).

Healthcare workers (HCWs) are considered to be at high risk for contracting and transmitting COVID-19 disease. The hospital-acquired COVID-19 rate for both inpatients and HCWs has been reported to be as high as 44% (Barranco et al., 2021). HCWs have also played a role in transmitting infection due to lack of knowledge about the disease, especially early during the pandemic when proper personal protective measures were not clearly defined (Karlsson and Fraenkel, 2020).

At King Faisal Specialist Hospital and Research Center in Riyadh, the majority of the patients are immunocompromised, placing them at higher risk for severe infection. For this study, serology testing of HCWs was performed at our Center in order to determine the prevalence of SARS-CoV-2 antibodies, as well as the associated risk factors, in the hope of implementing improved prevention and control measures.

## Methodology

This is an ongoing single-center observational prospective cohort study conducted at King Faisal Specialist Hospital and Research Center in Riyadh (the Center), Saudi Arabia, from June 2020 to December 2021. Serum SARS-COV-2 antibody prevalence will be determined at three different time points — at baseline, and then at 6 and 12 months later. This report represents the first set of antibodies — the baseline — which was collected over a 6-month period, from June to December, 2020. During this phase, none of the participants had received COVID-19 vaccine because it was not yet available in Saudi Arabia. Nasopharyngeal and throat swabs were used for COVID-19 PCR, and the PCR results were used to make a diagnosis of COVID-19 infection.

An email was sent to all Center employees, stating the aims and objectives of the study, and asking them to participate. All study subjects completed an online survey consisting of a set of variables, including: subject demographics; whether they were assigned to patients infected with SARS-CoV-2; whether they used personal protective equipment (PPE) as directed by the hospital infection control and hospital epidemiology department; if they had contact with infected employees, and the dates and duration of the contact; if they were diagnosed with COVID-19 disease; and any related symptoms. The duration of contact with infected employees was divided into two groups — less than 15 minutes and more than 15 minutes. Serum testing for the SARS-COV-2 antibody was performed after completing the survey. The Center's research ethical committee granted a waiver of informed consent for this study.

Participants were divided according to their occupation into clinical and nonclinical jobs, for further risk stratification. Clinical jobs included physicians, nurses, lab technicians, radiology technicians, respiratory therapists, pharmacists, and paramedics. Nonclinical jobs included social services, housing services, dietary and nutrition services, IT support, and administrative jobs. Work areas for nurses were classified into high- and low-risk areas. High-risk areas were COVID-19 units, the emergency department, and intensive care units. Low-risk areas were non-COVID-19 general wards, operating rooms, labor and delivery units, and the outpatient department. Participating employees were divided into three groups: group 1 took care of COVID-19 patients; group 2 had contact with a COVID-positive employee; and group 3 did not take care of COVID-19 patients and had no contact with infected employees.

### Molecular testing

RNA extraction was performed using Bioneer's ExiProgen™ RT-PCR kit and Kingfisher semi-automated flex purification system (Thermo Fisher Scientific) with 96 deep-well plates, following the manufacturer's recommendations. The Solgent DiaPlexQ™ Novel Coronavirus (2019-nCoV) Detection Kit was used for detection of ORF1a and the N gene of SARS-CoV-2, using Multiplex one-step qRT-PCR.

### Serology testing

Qualitative detection of serum antibodies to SARS-CoV-2 was performed using the Elecsys® Anti-SARS-CoV-2 kit (Roche Diagnostics) and reported as positive or negative serology. This assay uses a recombinant protein representing the nucleocapsid antigen in a double-antigen sandwich assay format, which favors detection of high-affinity antibodies against SARS-CoV-2.

A SARS-CoV-2 IgG assay was performed using the ARCHITECT i System (Abbott Diagnostics) to detect immunoglobulin class G (IgG) antibodies to the nucleocapsid protein of SARS-CoV-2 in serum from patients. The results of this testing were used for the sensitivity and specificity analyses.

### Statistical analysis

Subject demographics and risk factors were summarized using the mean, median, percentage, interquartile range, and standard deviation. A chi-square test was used to evaluate correlations between risk factors and COVID-19 disease. Univariate and multivariable logistic regression analyses were used to evaluate the odds of seroconverting between different groups. Sensitivity and specificity were calculated for IgG antibodies as a diagnostic tool for infection in HCWs. Statistical analyses were performed using IBM SPSS v.26. Values of *p* < 0.05 were considered statistically significant.

## Results

### Subject demographics

1076 subjects participated in this study, 647 (60.1%) women. The mean subject age was 38.4 ± 11.6 years (Table 1). 925 (85.9%) subjects were non-smokers (Table 1). Most subjects (775, 72%) had clinical jobs and were involved in direct patient care. The majority of these were nurses (379, 35%) or physicians (245, 22.8%). Of the nurses, 222 (58.58%) worked in low-risk areas and 157 (41.42%) worked in high-risk areas.

**Table 1:** Demographic characteristics of the 1076 subjects.

Characteristic	
Mean age, years (SD)	38.4 (11.6)
Female gender, *n* (%)	647 (60.13%)
Smoker, *n* (%)	151 (14.03%)
Job at KFSH&RC Clinical duties, *n* (%) Non-clinical duties, *n* (%)	775 (72.03%) 301 (27.97%)
Nurses’ area of coverage High risk, *n* (%) Low risk, *n* (%)	157 (41.42%) 222 (58.58%)
Subject group Group 1, *n* (%) Group 2, *n* (%) Group 3, *n* (%)	392 (36.4%) 463 (43%) 115 (10.7%)
Diagnosed with COVID-19, *n* (%)	265 (24.6%)
Symptomatic at diagnosis, *n* (%)	224 (84.5%)
Presenting symptoms, *n* (%) Headache Fever Sore throat Dry cough Diarrhea SOB	141 (62.9%) 131 (58.4%) 107 (47.8%) 97 (43.3%) 68 (30.3%) 43 (19.1%)
Positive serology (total antibody), *n* (%)	261 (24.3%)

### Risk group analysis

Of the 1076 subjects, 392 (36.4%) were caregivers for COVID-19 patients (group 1), 463 (43%) reported contact with infected employees (group 2), and 221 (20.5%) did not work with COVID-19 patients and had no contact with an infected employee (group 3). The majority of subjects were symptomatic at the time of diagnosis. Headache, fever, upper respiratory tract symptoms, and diarrhea were the most common presenting symptoms. 41 subjects (15.5%) reported no symptoms at the time of diagnosis.

There was a statistically significant association between taking care of COVID-19 patients and being diagnosed with COVID-19 disease (chi-square test, *p* = 0.046). There was no significant association between taking care of COVID-19 patients and being symptomatic at diagnosis (chi-square test, *p* = 0.079). 93 subjects (20.1%) who had contact with infected employees were diagnosed with COVID-19 disease, and 75 (80.6 %) of these individuals were symptomatic at the time of diagnosis. There was a significant association between being in contact with infected employees and being symptomatic at the time of diagnosis (chi-square test, *p* = 0.002). Among the employees in group 3, 115 (52%) were diagnosed with COVID-19 disease, of whom 64 (55.6%) were symptomatic at diagnosis.

The three employee risk groups were further evaluated. 743 (69.0%) of the subjects reported wearing proper PPE. PPE use was reported more frequently in group 1 than in group 2 (93.4% vs 81.4%, respectively) (Table 2). Most subjects in group 2 reported a contact duration of more than 15 minutes with infected employees (298, 64.4%). There was no association between the duration of contact with infected employees and diagnosis with COVID-19 disease (chi-square test, *p* = 0.224).

**Table 2:** Characteristics of the three risk groups; data are presented as *n* (%)

Group†	Exercised proper use of PPE	Diagnosed with COVID-19	Symptomatic at diagnosis	Positive serology* with prior diagnosis of COVID-19	Positive serology* with no prior diagnosis of COVID-19	Negative serology with prior diagnosis of COVID-19
Group 1 *N* = 392	366 (93.4%)	83 (21.2%)	52 (62.6%)	69 (83.1%)	15 (5.3%)	14 (16.8%)
Group 2 *N* = 463	377 (81.4%)	93 (20.1%)	75 (80.6%)	77 (82.7%)	10 (3.5%)	16 (17.2%)
Group 3 *N* = 221	NA	115	64 (55.6%)	95 (82.6%)	22 (20.7%)	20 (17.3%)

NA: not applicable

† There was an overlap between the groups in 27 subjects who took care of COVID-19 patients and were in contact with infected employees.

⁎ Serology included both IgG and IgM antibodies.

### IgG SARS-CoV-2 positivity

Among all 1076 participants, the overall seropositivity rate was 24.3% (Table 1). Of the HCWs assigned to COVID-19 patients (group 1), 71 (18%) were found to have positive IgG antibody results from their sera. There was no significant association between taking care of COVID-19 patients and having positive IgG antibodies (chi-square test, *p* = 0.215).

Of the subjects who had contact with infected employees, 68 (14.7%) were seropositive for IgG antibody. There was a significant association between being in contact with infected employees and having a positive IgG test (chi-square test, *p* < 0.001).

IgG antibody was assessed as a diagnostic tool for COVID-19 disease, demonstrating a sensitivity of 71.9% (95% CI: 66.4–77.30%) and specificity of 96.5% (95% CI: 95.04–97.69%), with a negative predictive value of 91.45% (95% CI: 89.80–92.86%) (Table 3). The positive likelihood ratio was 20.78 (95% CI: 14.33–30.14) and the negative likelihood ratio was 0.29 (95% CI: 0.24–0.35). This high negative predictive value for anti-nucleocapsid IgG makes it a good indicator for the absence of prior infection, with an accuracy of 90.55% (95% CI: 88.64–92.24%) (Table 3).

**Table 3:** Sensitivity and specificity of using IgG for the diagnosis of COVID-19

	Value	95% CI
Sensitivity	71.92%	66.04–77.30%
Specificity	96.54%	95.04–97.69%
Positive likelihood ratio	20.78	14.33–30.14
Negative likelihood ratio	0.29	0.24–0.35
Disease prevalence*	24.32%	21.78–27.01%
Positive predictive value*	86.98%	82.16–90.64%
Negative predictive value*	91.45%	89.80–92.86%
Diagnostic odds ratio*	90.55%	88.64–92.24%

⁎ These values are dependent on disease prevalence.

## Discussion

A nationwide study in Saudi Arabia reported a SARS-CoV-2 seropositivity rate of 2.3% among HCWs in different hospitals and regions in Saudi Arabia in May 2020, during the early stages of the pandemic (Alserehi et al., 2021). The highest rates of seropositivity were identified in the Makkah region (6.31%) and Al-Madinah region (4.55%) — hotspots of SARS-CoV-2 infection (Alserehi et al., 2021). A seropositivity rate among HCWs of 24.4% was reported in the Al-Madinah region from April to June, 2020 (Alharbi et al., 2020). In contrast, seropositivity rates of 2.36%, 6.3%, and 7.5% in healthy blood donors, non-COVID-19 patients, and HCWs, respectively, were reported from the Riyadh region from June to August, 2020 (Alharbi et al., 2021). The national seropositivity rate at the time was 11% (range: 1.78–24.45%) (Alharbi et al., 2021).

The baseline results of our planned prospective study, evaluating the prevalence and durability of SARS-CoV-2 antibody response in workers at a tertiary care hospital in Riyadh, Saudi Arabia from June to December, 2020, found an overall prevalence rate of 24.3% for this cohort. This demonstrated an increase in seropositivity in our HCWs with the progression of the pandemic. A potential contribution to this increase was the improper use of PPEs. Around 20% of group 2 subjects, who did not deliver care to COVID-19 patients, but were in contact with SARS-CoV-2-infected hospital staff, did not use masks when they were in contact with the infected staff. Universal masking for all hospital employees was implemented in early October 2020, 4 months after the start of this study. Including the time required to become proficient with this new policy, and also considering the incubation period of SARS-CoV-2 infection, these subjects were relatively unprotected for the majority of the study period. Implementation of PPE in developed, high-income countries has been shown to decrease the rate of conversion to SARS-CoV-2 seropositivity from 21.3% — a rate similar to ours — to 11.46%, a mean decline of 0.49% per day (Wang et al., 2020).

Testing of HCWs was performed at a New York City hospital from May to June, 2020, during an intense period of the pandemic in the city that coincided with PPE use by physicians. There was a higher rate of seropositivity among office staff (25.8%) and administrative HCWs (20.9%), compared with physicians (18.1%) (Purswani et al., 2021). These findings suggest that the proper use of PPE in a healthcare setting contributes to the control of disease spread (Purswani et al., 2021). Physicians in this study demonstrated differences in SARS-CoV-2 antibody seropositivity according to the zip code of their residence, possibly due to differences in disease prevalence in the city and reduced use of PPE when away from the hospital (Purswani et al., 2021).

Published reports of the prevalence of asymptomatic COVID-19 patients vary. The SARS-CoV-2 seropositive rate among HCWs in major hospitals, who underwent voluntary screening, ranged from zero to 7.5% in March and April of 2020 (Al-Zoubi et al., 2020; Treibel et al., 2020). Around a year into our study, 15.4% of the PCR-positive subjects we examined were asymptomatic at the time of diagnosis. These subjects were likely tested as part of the routine hospital screening protocol or as part of contact tracing. This higher rate of asymptomatic COVID-19 disease among HCWs was likely related to a higher rate of community transmission occurring at this later date, and to the introduction of routine PCR testing.

Of our asymptomatic SARS-CoV-2-seropositive subjects, 3.6% had no prior diagnosis of COVID-19 infection. In their national survey, Alhrabi et al. estimated that the seropositive rate for individuals in Riyadh was six times higher than that for SARS-CoV-2 infections diagnosed using PCR testing (2021). This further highlights the importance of asymptomatic transmission of SARS-CoV-2 and its potential for causing hospital outbreaks. Investigation of a hospital outbreak in Portugal, in the summer of 2020, revealed 27 HCWs and 21 patients who tested positive for SARS-CoV-2; 24 (88.9%) of the HCWs were asymptomatic at the time of diagnosis (Borges et al., 2021). Genomic and epidemiological analysis revealed that the outbreak was caused by HCW–patient and HCW–HCW transmission (Borges et al., 2021). Identification of the pattern of spread led to the introduction or emphasis of several safety measures, including frequent hand cleansing, maintenance of a safe physical distance, and mandatory use of respiratory protection. These findings have particular relevance to the emergence of different SARS-CoV-2 variants thought to be more transmissible and possibly associated with higher mortality (Challen et al., 2021, Davies et al., 2021).

There were several limitations to this study. Recruitment was slow — it took 6 months to accrue 1076 subjects. During the study period, IgM antibody tests were not performed for all employees due to shortage of kits during the pandemic; however, IgG kits were available throughout the study period. This presented findings were for volunteers, who may not have been representative of the overall population working at the Center. No data regarding community exposure were available. These factors could have affected the high rate of seropositivity seen in this study. Subject medical data, which could have affected the rate of seropositivity or development of symptoms, were not available.

IgG testing revealed that SARS-CoV-2 serological testing was highly sensitive and specific, and had a high negative predictive value in the diagnosis of COVID-19 disease. These findings may not help with disease containment, because IgG tends to appear later in the course of the disease. In contrast, PCR testing yields an early positive result (Murad et al., 2021). Early diagnosis can lead to the prompt introduction of isolation and quarantine practices. In areas with limited COVID-19 vaccine availability, these findings may be used to prioritize vaccination for seronegative HCWs. Patient risk assessment may be performed using IgG testing, because the presence of anti-spike or anti-nucleocapsid SARS-CoV-2 IgG antibodies has been reported to be associated with a substantially reduced risk of reinfection for at least 6 months (Lumley et al., 2021).

## Conclusions

A baseline analysis of SARS-CoV-2 seropositivity in HCWs at a large tertiary care hospital in Riyadh was performed as the first part of a prospective study of HCWs. The reported seropositivity was 24.3% — higher than that of other hospitals in Riyadh. IgG testing was very useful in the detection of previous SARS-CoV-2 infection, because it has high negative predictive value.
